# Propofol ameliorates ischemic brain injury by blocking TLR4 pathway in mice

**DOI:** 10.1515/tnsci-2022-0238

**Published:** 2022-09-01

**Authors:** Kazuha Mitsui, Masakazu Kotoda, Sohei Hishiyama, Ayasa Takamino, Sho Morikawa, Tadahiko Ishiyama, Takashi Matsukawa

**Affiliations:** Department of Anesthesiology, Faculty of Medicine, University of Yamanashi, 1110 Shimokato, Chuo, Yamanashi 409-3898, Japan

**Keywords:** propofol, toll-like receptor 4, ischemic brain injury

## Abstract

Ischemic brain injury is one of the most serious perioperative complications. However, effective preventative methods have not yet been established. This study aimed to investigate whether propofol has neuroprotective effects against ischemic brain injury, with a specific focus on Toll-like receptor 4 (TLR4). Focal brain ischemia was induced via a combination of left common carotid artery occlusion and distal left middle cerebral artery coagulation in mice. Either propofol (10 mg/kg) or vehicle was intravenously injected 10 min prior to the induction of brain ischemia in wild-type and TLR4 knockout mice. Infarct volume, pro-inflammatory cytokine expression, inflammatory cell infiltration, and neurobehavioral function were assessed. Propofol administration significantly reduced infarct volume in wild-type mice (26.9 ± 2.7 vs 15.7 ± 2.0 mm^3^ at day 7), but not in TLR4 knockout mice. Compared with the control mice, the propofol-treated wild-type mice exhibited lower levels of IL-6 (0.57 ± 0.23 vs 1.00 ± 0.39 at 24 h), and smaller numbers of TLR4-expressing microglia in the penumbra (11.7 ± 3.1 vs 25.1 ± 4.7 cells/0.1 mm^2^). In conclusion, propofol administration prior to ischemic brain insult attenuated brain injury by blocking the TLR4-dependent pathway and suppressing pro-inflammatory cytokine production.

## 1 Background

Ischemic brain injury is a serious disease that compromises people’s lives and health [1] and is one of the most concerning perioperative complications. There are various causes including hypotension, hypoxia, arrhythmia, systemic inflammation, and blood loss due to surgery/anesthesia that jeopardize cerebral circulation. Surprisingly, in recent studies using magnetic resonance imaging, the incidence of ischemic brain injury associated with noncardiac surgery was as high as 10% in patients with cardiovascular risk factors [2], and thrombotic stroke is the most common type of this complication [3]. The occurrence of ischemic brain injury largely impedes recovery from surgery and is associated with an eightfold increase in perioperative mortality [4]. Furthermore, a recent prospective cohort study indicates that even covert ischemic brain injuries significantly increase the risk of long-term cognitive decline and can impair quality of life [5].

However, promptly detecting acute ischemic brain injury is sometimes challenging due to the remaining anesthetic effects or the use of postoperative opioids and sedative drugs. Consequently, only a limited number of patients receive reperfusion therapy during the acute phase. Therefore, preventative strategies for minimizing ischemic brain injury are vital. In addition, the effects of commonly used anesthetic drugs on the ischemic brain and its underlying mechanisms are relevant to research targets. Given these considerations, continuous efforts have been made in recent decades to elucidate the effects of anesthetic drugs on ischemic brain injury, and volatile anesthetic drugs have been shown to exert preconditioning and neuroprotective effect via activation of mitochondrial adenosine triphosphate-sensitive potassium channels [6,7]. Some earlier studies using animal models of ischemic stroke or cell culture have also suggested neuroprotective effects of propofol [8,9,10]. However, the evidence is still limited and controversial [11], and the mechanisms underlying the possible neuroprotective effects of propofol remain unclear. Several recent studies suggest that propofol blocks Toll-like receptor 4 (TLR4) is the key mediator of inflammation and suppresses inflammatory cytokine production [12,13,14]. Given that inflammation is a crucial factor in secondary injury after brain ischemia and that TLR4 plays a pivotal role in the inflammatory response in the ischemic brain [15,16], the TLR4-dependent pathway may be involved in the possible neuroprotective effects of propofol against ischemic brain injury.

Thus, we tested the hypothesis that propofol attenuates ischemic brain injury via inhibition of the TLR4-dependent pathway and suppression of consequent inflammatory cytokine production. The primary outcome of this study was infarct volume; the secondary outcomes were pro-inflammatory cytokine mRNA expression levels, the number of TLR4-expressing microglia in the penumbra, and neurological function.

## 2 Materials and methods

### 2.1 Animals

Male C57BL/6 mice (8 weeks old) were purchased from Japan SLC (Tokyo, Japan). Two breeding pairs of TLR4 knockout mice with a C57BL/6 genetic background were purchased from Oriental Bio Service (Kyoto, Japan), and their male offspring mice (10–14 weeks old) were used in the study. All mice were group-housed at 23°C ± 2°C with free access to standard food and water and a 12-h light/dark cycle. All experiments were performed between 09:00 and 17:00 under normal room light and temperature (23°C ± 2°C) conditions.


**Ethical approval:** All experiments were conducted in accordance with the National Institutes of Health guidelines for the care and use of laboratory animals. The experimental protocol was reviewed and approved by the University of Yamanashi Animal Care Committee.

### 2.2 Ischemic brain injury

Focal brain ischemia was induced via a combination of permanent left common carotid artery occlusion and distal left middle cerebral artery (MCA) coagulation [17]. Briefly, mice were anesthetized with 2–3% isoflurane and placed in a dorsal position. The left common carotid artery was isolated and ligated via ventral middle neck incision. Mice were then placed in the lateral position, and a 2 mm burr-hole craniectomy was performed with a microdrill (Ideal Microdrill; Bio Research, Nagoya, Japan) between the left orbit and the left ear. The distal left MCA was exposed and coagulated using a small vessel cauterizer (Fine Science Tools, Inc., CA, USA) followed by a transection of the artery. During the surgery, rectal temperature was maintained at 37°C ± 0.5°C with a thermostat-regulated heating pad. Brains were removed 24 h after the induction of ischemic brain injury for real-time polymerase chain reaction (PCR) or immunofluorescence or at 7 days for measurement of infarct volume.

### 2.3 Propofol treatment

Propofol (10 mg/kg, 1% Diprivan, Aspen Japan, Tokyo, Japan) diluted with fat emulsion by 10 times (10 µL/g, Intralipos, Otsuka Pharmaceutical, Tokyo, Japan) was administered via the tail vein 10 min prior to MCA occlusion. In control mice, an equal volume of the fat emulsion alone was administered. The dose was chosen based on an earlier study, in which propofol produced neuroprotection against ischemic brain injury in mice [18].

### 2.4 Measurement of infarct volume

Seven days after MCA occlusion and after hemodynamic measurements, mice were deeply anesthetized with 5% isoflurane and euthanized via cervical dislocation. Brains were removed and cut into 1-mm-thick coronal sections. The brain slices were immersed in 2% 2,3,5-triphenyltetrazolium chloride (Sigma Aldrich, St. Louis, MO, USA) at 37°C for 15 min in a dark room. The infarct area was traced and measured using image analysis software (ImageJ, National Institutes of Health, Bethesda, MD, USA) by an individual who was blinded to the grouping and study design. To correct for the contribution of edema, the infarct area was calculated as follows: total ipsilateral hemisphere – infarct region [19]. Total infarct volume was calculated as the sum of all infarct areas multiplied by section thickness.

### 2.5 Hemodynamic measurements

Heart rate and arterial blood pressure were measured non-invasively using a tail-cuff monitor (Softron, Tokyo, Japan) to evaluate the effects of propofol on hemodynamics. Values were recorded 1 h before MCA occlusion (baseline), 10 min after the injection of either propofol or the control solution, 1 h after MCA occlusion, and 7 days after MCA occlusion.

### 2.6 Blood gas analysis

Blood gas analysis was performed to evaluate pH, PCO_2_, PO_2_, and glucose levels (i-STAT 300 F, Abbot Co., Abbot Park, IL, USA). Blood samples were collected before induction of MCA occlusion (baseline), 1 h after the injection of either propofol or the control solution, and 7 days after MCA occlusion.

### 2.7 Real-time PCR

Real-time PCR was used to measure mRNA expression levels of interleukin (IL)-6, IL-1β, and tumor necrosis factor α (TNF-α). The brain was the target mRNAs from the brain slices of propofol-treated wild-type mice that were compared with those of control wild-type mice. The RNeasy Mini Kit (Qiagen, Hilden, Germany) was used for the extraction of total mRNA from brain slices. About 1µg of mRNA was reverse transcribed into complementary DNA with a QuantiTect Reverse Transcription Kit (Qiagen, Hilden, Germany). PCR was performed on a StepOne^TM^ real-time PCR system (Life Technologies, Carlsbad, CA, USA) using the PowerSYBR^®^ Green PCR Master Mix and corresponding primers to quantify target genes (Table S1). The relative changes were expressed as a ratio to glyceraldehyde-3-phosphate dehydrogenase (GAPDH) mRNAs of the same sample. The data were analyzed using the 2^−ΔΔCT^ method. The 2^−ΔΔCT^ value of the target transcript from each mouse was normalized with those of the control group mice as 1.0. The normalized 2^−ΔΔCT^ values derived from the two groups were then compared.

### 2.8 Immunofluorescence

The mouse brains were fixed with 4% paraformaldehyde in phosphate-buffered saline (PBS) for 6 days and cut into 1mm-thick sections using a slicer (BD80 HS, Leica, Bensheim, Germany). Prior to immunofluorescence, CUBIC tissue clearing [20] was performed to identify ischemic core and surrounding penumbra. The brain sections were washed three times with PBS and then immersed in 50% CUBIC-L (T3740, Tokyo Chemical Industry, Tokyo, Japan) overnight with shaking at 40°C, followed by immersing in 100% CUBIC-L with shaking at 40°C for 3 days, replacing 100% CUBIC-L with new solution every day. Then, the brain sections were washed three times with PBS and stained with 0.5% TritonX-100 (12967-32, NACALAI TESQUE, INC., Kyoto, Japan) in PBS and the following primary antibodies for 5 days in the dark with shaking at room temperature: FITC-conjugated rat anti-TLR4 (1:25, sc-13591, Santa Cruz, CA, USA) and Alexa Fluor 594-conjugated rat anti-CD11b (1:100, 101254, BioLegend, CA, USA). The stained-brain sections were washed three times with PBS and then post-fixed with 1% formaldehyde in PBS overnight. After washing twice with PBS, to complete CUBIC tissue clearing, the brain sections were immersed in 50% CUBIC-R (T3741) overnight followed by 100% CUBIC-R overnight with shaking at room temperature. Finally, the sections were photographed using a confocal microscope (A1R HD25, Nikon, Tokyo, Japan).

TLR4 + CD11b + double-positive cells in the penumbra, defined as the region immediately adjacent to the infarct area, were counted in 400× magnification microscopic fields. For each animal, ten 0.1 mm^2^ areas within the cortex were randomly chosen, and the number of TLR4 + CD11b + double-positive cells was automatically counted using Image J software. Ten readouts per animal were averaged.

### 2.9 Neurological evaluation

Neurological function was assessed using the neurological deficit scores (0: no deficit; 1: flexion of the torso; 2: spontaneous circling; 3: longitudinal circling or leaning; 4: no spontaneous movement; 5: death), lateral push test, and body asymmetry test at baseline and 7 days after the induction of ischemic brain injury. The lateral push test and body asymmetry test were performed as previously described [21,22].

### 2.10 Statistical analysis

Statistical analysis was performed using Prism 8 software (GraphPad Software, San Diego, CA, USA). The collected data were assessed for the normal distribution and equal variance using the Shapiro–Wilk and *F* test, respectively. Infarct volumes, numbers of TLR4 + CD11b + double-positive cells, and body asymmetry test results were analyzed using two-tailed Student’s *t* test; cytokine expression levels and neurological deficit scores were analyzed using Mann–Whitney *U* test, based on the results of the Shapiro–Wilk and *F* tests. Heart rate, blood pressure, and blood gas parameters (pH, PCO_2_, PO_2_, and glucose) were analyzed using the two-way analysis of variance for repeated measures; the lateral push test results were analyzed using Chi-square test. For infarct volumes and cytokine expression levels, the sample size was calculated to detect significance with 95% confidence, assuming alpha of 0.05 and power of 0.8 (G*Power 3.1.9.3). Values are presented as mean ± standard error of mean for the infarct volumes and numbers of cells, as median ± quantile for cytokine mRNA expression levels, and as mean ± standard deviation for heart rate, blood pressure, and blood gas parameters. *p*-values of less than 0.05 were considered statistically significant.

## 3 Results

### 3.1 Propofol reduced cerebral infarct volume

**Figure 1:**
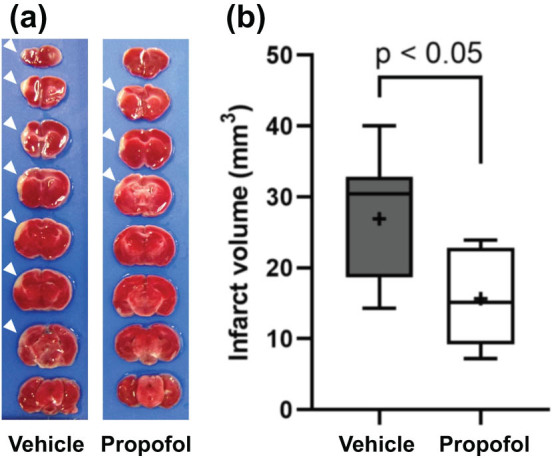
Effect of propofol pretreatment on infarct volume: (a) Staining for 2,3,5-triphenyltetrazolium chloride in representative 1-mm-thick coronal sections. Arrowheads indicate the infarct area (white). (b) Infarct volumes 7 days after induction of ischemic brain injury. Mice in the propofol group exhibited smaller infarct volumes.

To investigate the effects of propofol on ischemic brain injury, 8- to 9-week-old wild-type mice were treated with either propofol or 10% fat emulsion (control) 10 min before the induction of ischemic brain injury. Propofol-treated mice exhibited significantly smaller infarct volumes than control mice 7 days after ischemic brain injury (26.9 ± 2.7 vs 15.7 ± 2.0 mm^3^, *n*  =  10 each, *p*  <0.05, Figure 1). Propofol treatment did not affect the hemodynamic and blood gas parameters and neurological function (neurological deficit scores, baseline and day 7: 0.0 ± 0.0 vs 0.0 ± 0.0, *p*  > 0.99; lateral push test, baseline and day 7: 0.0 ± 0.0 vs 0.0 ± 0.0, *p*  > 0.99; body asymmetry test, baseline: 6.0 ± 5.4 vs 1.0 ± 6.4, *p*  = 0.56, day 7: 9.0 ± 12.5 vs 26.0 ± 9.1, *p*  = 0.29, all *n*  =  10 each, Table 1).

**Table 1:** Results of hemodynamic measurements and blood gas analysis

		Wild-type + vehicle	Wild-type + propofol	TLR4KO + vehicle	TLR4KO + propofol
Heart rate (bpm)	Baseline	443 ± 54	423 ± 36	445 ± 64	465 ± 50
	10 min Before MCAO	428 ± 29	448 ± 63	388 ± 26	367 ± 40
	1 h After MCAO	442 ± 77	429 ± 84	375 ± 27	395 ± 20
	7 Days after MCAO	473 ± 74	468 ± 64	421 ± 37	454 ± 41
Mean blood pressure (mmHg)	Baseline	77 ± 17	82 ± 9	79 ± 8	80 ± 8
	10 min Before MCAO	74 ± 12	77 ± 9	81 ± 8	74 ± 12
	1 h After MCAO	87 ± 14	76 ± 12	83 ± 4	80 ± 16
	7 Days after MCAO	86 ± 8	80 ± 10	85 ± 8	83 ± 16
pH	Baseline	7.34 ± 0.06	7.28 ± 0.06	7.33 ± 0.05	7.33 ± 0.06
	1 h After MCAO	7.30 ± 0.01	7.31 ± 0.04	7.30 ± 0.04	7.31 ± 0.04
	7 Days after MCAO	7.30 ± 0.05	7.35 ± 0.02	7.34 ± 0.02	7.33 ± 0.03
PCO_2_ (mmHg)	Baseline	34.8 ± 5.7	38.9 ± 4.7	34.6 ± 1.4	36.4 ± 4.6
	1 h After MCAO	45.4 ± 1.2	44.2 ± 3.2	45.0 ± 3.8	46.3 ± 9.2
	7 Days after MCAO	44.0 ± 3.3	39.6 ± 6.3	41.6 ± 3.8	38.8 ± 5.9
PO_2_ (mmHg)	Baseline	59.4 ± 8.8	70.6 ± 10.9	64.0 ± 4.6	63.8 ± 5.5
	1 h After MCAO	64.0 ± 13.0	64.0 ± 6.7	70.4 ± 6.3	68.2 ± 5.4
	7 days After MCAO	53.6 ± 7.9	55.0 ± 6.4	64.2 ± 9.9	61.0 ± 5.1
Glucose (mg/dL)	Baseline	252.6 ± 46.8	264.6 ± 35.1	257.8 ± 67.5	255.8 ± 15.0
	1 h After MCAO	251.0 ± 42.6	204.2 ± 40.5	246.2 ± 33.5	229.8 ± 19.8
	7 Days after MCAO	254.8 ± 31.3	227.2 ± 22.2	209.4 ± 62.4	191.2 ± 42.2

There were no significant differences in heart rate, blood pressure, and blood gas parameters among groups. (All parameters: *p* > 0.05.). Data were presented as mean ± SD. MCAO, middle cerebral artery occlusion; TLR4KO, toll-like receptor 4 knockout.

### 3.2 Propofol treatment reduced pro-inflammatory cytokine expressions after ischemic brain injury

**Figure 2:**
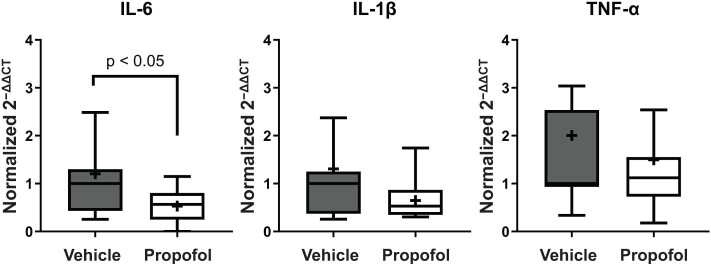
Assessment of pro-inflammatory expression levels 24 h after the induction of ischemic brain injury. The 2^−ΔΔCT^ value of the target transcript from each mouse was normalized with the median 2^−ΔΔCT^ value from the control wild-type mice as 1.0. Mice in the propofol group exhibited lower levels of inflammatory cytokine expression.

To investigate the possible involvement of anti-inflammatory action of propofol in the neuroprotective effect observed, mRNA expression of pro-inflammatory cytokines was measured 24 h after ischemic brain injury. As shown in Figure 2, the propofol-treated mice exhibited lower mRNA expression levels of pro-inflammatory cytokines (IL-6: 0.57 ±  0.23 vs 1.00 ±  0.39, *p*  <  0.05, IL-1β: 0.53 ±  0.24 vs 1.00  ±  0.36, *p* = 0.087, *n*  =  15 each) compared with the control mice.

### 3.3 Propofol decreased the number of TLR4-expressing microglia in the penumbra

**Figure 3:**
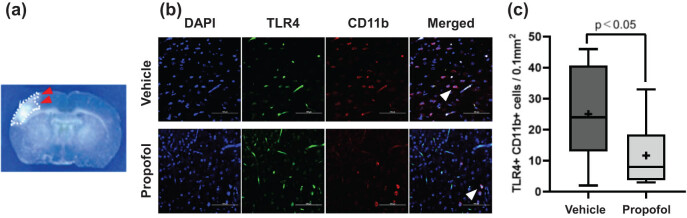
Assessment of the number of TLR4-expressing microglia in the penumbra: (a) the representative image of the brain slice after CUBIC tissue cleaning. The red arrowheads indicate the penumbra, defined as the region immediately adjacent to the infarct area (encircled with white dashed line), used for immunofluorescence assay. (b) Immunofluorescence staining of DAPI (blue), TLR4 (green), CD11b-positive microglia (red), and merged images were presented. The white arrowheads indicate TLR4 + CD11b + double-positive cells in the merged images. (c) Compared with the control mice, the propofol-treated mice had significantly smaller numbers of TLR4-expressing microglia in the penumbra.

Immunofluorescence targeting TLR4 and microglia was performed to assess whether the propofol treatment influenced TLR4 expression or infiltration of microglia in the penumbra. As shown in Figure 3, mice treated with propofol had smaller numbers of TLR4-expressing microglia in the penumbra, compared with the control mice (11.7 ± 3.1 vs 25.1 ± 4.7 cells/0.1 mm^2^, *n* = 10 each, *p*  <  0.05).

### 3.4 Neuroprotective effects of propofol were abolished by depletion of the TLR4-dependent pathway

**Figure 4:**
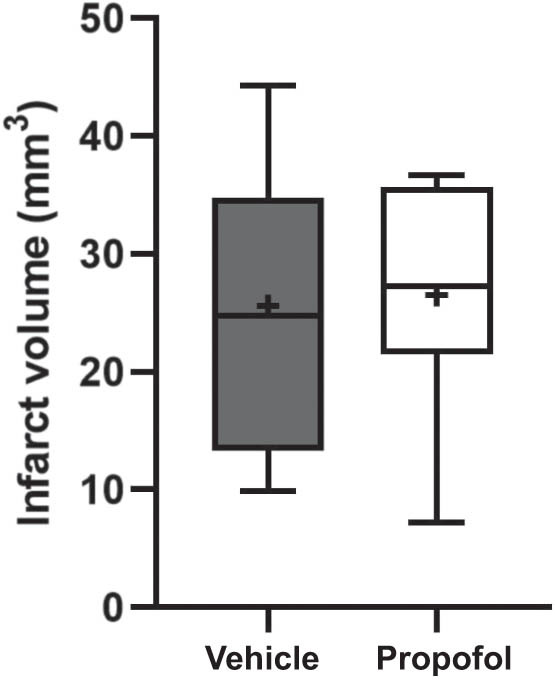
Effect of propofol on infarct volumes in Toll-like receptor-4 (TLR4) knockout mice. Infarct volumes were assessed 7 days after the induction of ischemic brain injury. There was no significant difference in infarct volumes between the propofol-treated TLR4 knockout mice and control-treated TLR4 knockout mice.

In experiments testing the effects of propofol treatment against ischemic brain injury in TLR4 knockout mice, there was no significant difference in the infarct volume between the propofol-treated TLR4 knockout mice and control-treated TLR4 knockout mice (25.6 ± 3.8 vs 26.5 ± 3.1 mm^3^, *n* = 10 each, *p* = 0.91, Figure 4). Similar to the experiments using wild-type mice, hemodynamic and blood gas parameters and neurological function were not significantly different between the groups (neurological deficit scores, baseline and day 7: 0.0 ± 0.0 vs 0.0 ± 0.0, *p*  > 0.99; lateral push test, baseline and day 7: 0.0 ± 0.0 vs 0.0 ± 0.0, *p*  > 0.99; body asymmetry test, baseline: 7.5 ± 3.1 vs 11.1 ± 8.9, *p*  = 0.72, 7 day after: −10.0 ± 6.5 vs −1.1 ± 2.0, *p*  = 0.19, all *n* = 10 each, Table 1).

## 4 Discussion

In the present study, we found that propofol significantly reduced infarct volume after ischemic brain insult in wild-type mice but not in TLR4-knockout mice. The reduced infarct volume was associated with reduced pro-inflammatory cytokine expressions and TLR4-expresing microglia in the penumbra. These results indicated that propofol exerts neuroprotective effects via its anti-inflammatory capacity, specifically the inhibitory effect on TLR4. Since the hemodynamic and blood gas parameters were not affected by the propofol administration, the effects of propofol on cardiovascular and respiratory function and blood sugar levels were not likely to be involved in the neuroprotective effects of propofol observed in the present study.

The immune system is promptly activated once ischemic brain injury occurs [23]. Among various inflammatory mediators, TLR is considered crucial in innate immune system as a first-line defense and mediator of inflammation [24,25]. Exogenous/endogenous TLR ligands such as heat shock proteins, fibrinogen, and components of the extracellular matrix are upregulated by ischemic brain insult. Consequently, those ligands then activate TLR4 [26,27], the TLR shown to be the pivotal inflammatory mediator in the pathogenesis of ischemic brain injury [15,16]. It has been reported that while activation of TLR4 exacerbates cerebral infarction [15], inhibition of the receptor suppresses pro-inflammatory responses and attenuates brain injury [16].

In the current study, the neuroprotective effect of propofol against ischemic brain injury was abrogated by depletion of TLR4, indicating that the TLR4-dependent pathway was substantially involved in the mechanism underlying that neuroprotective effect. The results of the current study are concordant with recent *in vivo* studies in which propofol suppressed inflammatory cytokine production via inhibition of TLR4-dependent pathways in various disease models, including lung [12], liver [13], and gastric injuries [14], and asthma [28]. Several *in vitro* studies using lipopolysaccharide-treated microglia [29], macrophages [30], spinal astrocytes [31], and alveolar epithelial cells [32] also suggest that the anti-inflammatory action of propofol involves blocking the TLR4-dependent pathway and consequent pro-inflammatory cytokine production.

Our result showed significantly lower IL-6 mRNA expression levels in the brains of propofol-treated wild-type mice than in the brains of control mice, which is consistent with an earlier study in which there was a correlation between infarct volume and IL-6 mRNA expression in the brain [33] Among various pro-inflammatory cytokines, IL-6 plays pivotal roles in local inflammation and cytotoxicity after ischemic brain injury and is involved in the mechanism underlying the expansion of ischemic brain injury [34,35]. Blockade of IL-6 receptors has been shown to reduce infarct volume and improve cognitive function in an experimental model of ischemic stroke [36]. These earlier studies are concordant with the suppression of IL-6 by propofol observed in the current study after ischemic brain insult, as well as the reduction of infarct volume.

Our immunofluorescence study demonstrated that the pre-treatment with propofol reduced the number of TLR4-expressing microglia in the penumbra. Recent studies have reported that microglia are involved in the exacerbation of cerebral infarction [37,38,39]. TLR4-expressing microglia release pro-inflammatory cytokines [37], which causes inflammation in the penumbra and aggravates the brain injury. On the other hand, propofol has been shown to inhibit TLR4 upregulation in microglia [40]. Another previous study reported that propofol has neuroprotective effects against ischemic stroke by suppressing microglia [41]. Our real-time PCR and immunofluorescence results demonstrated that the propofol treatment decreased the expression level of IL-6 mRNA and the number of TLR4-expressing microglia after cerebral ischemia. Based on the collective results of the previous studies and the current study, it is reasonable to surmise that propofol exerts neuroprotection against ischemic brain injury by blocking TLR4 and suppressing consequent production of pro-inflammatory cytokines, particularly IL-6, from microglia in the penumbra.

If ischemic brain injury occurs during general anesthesia, the consequences are tragic. The clinical importance of the present study is that propofol administration prior to ischemic insult may have the potential to protect against ischemic brain injury, presumably by blocking the TLR4-dependent pathway in microglia in the penumbra. This understanding of the mechanism underlying the neuroprotective effect of propofol against ischemic brain injury may lead to a novel strategy to prevent exacerbation of ischemic brain injury during general anesthesia.

This study has some limitations. First, we did not find neurological improvement in the propofol-treated mice, possibly because of the relatively small infarct volumes and low sensitivities of the neurological assays we used in the current study. Although infarct volume is clinically relevant and was the primary outcome of this study, further studies with more sensitive neurobehavioral evaluation are needed to elucidate the effects of propofol on neurobehavioral function after ischemic brain injury. Second, we only used relatively young male mice. The neuroprotective effects of propofol should be tested in aged mice and female mice at different menopausal stages because those biological variables can affect the outcomes of ischemic brain injury. Third, we used only GAPDH as a reference gene for the real-time PCR analysis. Although this gene is widely used as a reference gene for evaluating inflammatory responses after ischemic stroke in mice, it is indicated that hypoxia upregulates GAPDH mRNA expression [42], which could have influenced the interpretation of the data. Lastly, we unexpectedly observed similar infarct sizes between TLR4 KO and wild-type mice. This observation is not consistent with the findings of previous studies that reported reduced infarct size by TLR4 gene knockout [43,44]. This may be explained by the compensatory upregulation of other related genes following a genetic depletion of a certain gene [45,46]. The TLR4 knockout could have induced the upregulation of other inflammatory pathways, such as TLR2 pathway, at least in our model. Another possible explanation would be that the first experiment (wild-type mice with/without propofol) and the second experiment (TLR4 knockout mice with/without propofol) were separately conducted. The ages of the mice used in the experiments were different (WT: 8 weeks old; TLR4 KO: 10–14 weeks old due to the breeding capacity). The wild-type animals were purchased, while the knockout mice were bred in our laboratory. Therefore, the infarct volume data from these two experiments might not be directly comparable. In this study, propofol pretreatment significantly attenuated brain infarction in the WT mice, but not in the TLR4 KO mice, indicating the involvement of the TLR4 pathway in the neuroprotective effects of propofol.

## 5 Conclusions

Propofol administration prior to ischemic brain insult attenuated brain injury by blocking the TRL4-dependent pathway and suppressing pro-inflammatory cytokine production. This understanding of the mechanism underlying the neuroprotective effect of propofol against ischemic brain injury may lead to a new strategy to prevent exacerbation of cerebral infarction during general anesthesia.

## List of abbreviations

IL-1β: interleukin-1β
IL-6: interleukin-6
MCA: middle cerebral artery
PCR: polymerase chain reaction
TLR4: Toll-like receptor 4
TNF-α: tumor necrosis factor-α

## Supplementary Material

Supplementary Table
