# Supplementary material for: Propofol ameliorates ischemic brain injury by blocking TLR4 pathway in mice
Source: Transl Neurosci. 2022 Sep 1;13(1):246–54. doi: 10.1515/tnsci-2022-0238 (PMC9438965; doi:10.1515/tnsci-2022-0238)
Supplement: Supplementary Table [file tnsci-2022-0238-sm.pdf]

Supplementary material

Table S1: Primers used for real-time PCR

Primers	Sequence
GAPDH-Forward	TGGCAAAGTGGAGATTGTTGCC
GAPDH-Reverse	AAGATGGTGATGGGCTTCCCG
IL-1β-Forward	GTAATGAAAGACGGCACACC
IL-1β-Rreverse	TACCAGTTGGGGAACTCTGC
IL-6-Forward	TCCAGTTGCCTTCTTGGGAC
IL-6-Reverse	GTGTAATTAAGCCTCCGACT
TNF-α-Forward	GCTTTCCGAATTCACTGGAG
TNF-α-Reverse	TTGCACCTCAGGGAAGAATC

GAPDH, glyceraldehyde-3-phosphate dehydrogenases; IL, interleukin; PCR, polymerase chain reaction; TNF, tumor necrosis factor.
